# Effect of Thoracic Connective Lesion on Inter-Leg Coordination in Freely Walking Stick Insects

**DOI:** 10.3389/fbioe.2021.628998

**Published:** 2021-04-20

**Authors:** Miriam Niemeier, Manon Jeschke, Volker Dürr

**Affiliations:** ^1^Department of Biological Cybernetics, Faculty of Biology, Bielefeld University, Bielefeld, Germany; ^2^Center for Cognitive Interaction Technology, Bielefeld University, Bielefeld, Germany

**Keywords:** walking, leg coordination, locomotion, neural coupling, load transfer

## Abstract

Multi-legged locomotion requires appropriate coordination of all legs with coincident ground contact. Whereas behaviourally derived coordination rules can adequately describe many aspects of inter-leg coordination, the neural mechanisms underlying these rules are still not entirely clear. The fact that inter-leg coordination is strongly affected by cut thoracic connectives in tethered walking insects, shows that neural information exchange among legs is important. As yet, recent studies have shown that load transfer among legs can contribute to inter-leg coordination through mechanical coupling alone, i.e., without neural information exchange among legs. Since naturalistic load transfer among legs works only in freely walking animals but not in tethered animals, we tested the hypothesis that connective lesions have less strong effects if mechanical coupling through load transfer among legs is possible. To do so, we recorded protraction/retraction angles of all legs in unrestrained walking stick insects that either had one thoracic connective cut or had undergone a corresponding sham operation. In lesioned animals, either a pro-to-mesothorax or a meso-to-metathorax connective was cut. Overall, our results on temporal coordination were similar to published reports on tethered walking animals, in that the phase relationship of the legs immediately adjacent to the lesion was much less precise, although the effect on mean phase was relatively weak or absent. Lesioned animals could walk at the same speed as the control group, though with a significant sideward bias toward the intact side. Detailed comparison of lesion effects in free-walking and supported animals reveal that the strongest differences concern the spatial coordination among legs. In free walking, lesioned animals, touch-down and lift-off positions shifted significantly in almost all legs, including legs of the intact body side. We conclude that insects with disrupted neural information transfer through one connective adjust to this disruption differently if they experience naturalistic load distribution. While mechanical load transfer cannot compensate for lesion-induced effects on temporal inter-leg coordination, several compensatory changes in spatial coordination occur only if animals carry their own weight.

## Introduction

Adaptive, coordinated walking requires appropriate and simultaneous control of multiple legs (e.g., Graham, 1985; Dürr et al., 2018; Ritzmann and Zill, 2019). While it is clear that the interplay of rhythmic movements of all legs is monitored and controlled by neuronal circuits and proprioceptive systems (Tuthill and Wilson, 2016), neurophysiological and behavioural evidence on leg coordination support slightly different weighting of the relative importance of proprioceptive feedback in the generation of a gait.

Neurophysiological evidence from insects suggests that temporal patterning of activity in leg motor nerves arises from local neuronal networks of each leg, convened into central pattern generators (Bidaye et al., 2018), whose action can be adjusted by both intra- and inter-limb sensory feedback (Büschges, 2005). Accordingly, the gait originates from central neural network dynamics that is adjusted by proprioceptive input. In comparison, behavioural evidence, particularly from stick insects and crayfish, has been summarised in a set of coordination rules that describe the pairwise interaction between neighbouring legs (Cruse, 1990). In various software and hardware models of insect locomotion (e.g., Cruse et al., 1998; Dürr et al., 2019; Schilling and Cruse, 2020) these coordination rules have been implemented as sensory-motor feedback mechanisms. As the pairwise coupling through these feedback mechanisms dominates the execution of each step cycle, the gait does not originate from central neural network dynamics but emerges from distributed interaction of the body and its environment (Schilling et al., 2013). While this allows for several aspects of behavioural flexibility through de-centralised inter-leg coordination (Dürr et al., 2018), the neuronal mechanisms that underlie pairwise inter-leg coupling are not entirely clear.

The present study aims to quantify the contribution of local, load-dependent sensory feedback in insect walking without ipsilateral neural coordination. Experiments on tethered walking stick insects showed that inter-leg coordination is strongly affected by cutting thoracic connectives. Following connective lesions, animals showed shifted touch-down and lift-off positions of the tarsi and temporally uncoordinated step cycles of neighbouring legs (Dean, 1989). This strongly suggested that neural information exchange among legs is important. However, as rhythmic movement persisted in the leg posterior to the lesion, the generation of a local step cycle was still possible without neural input from the anterior hemi-ganglion.

More recently, experiments on freely walking stick insects showed that step cycles of ipsilateral neighbouring legs can be coordinated due to mechanical coupling alone (Dallmann et al., 2017). This study suggests that load transfer among legs generates sensory information about unloading that can be registered by campaniform sensilla (Zill et al., 2004) which, in turn, drive local reflex circuits involved in inter-leg coordination. Similar sensorimotor mechanisms were also discussed in cockroaches (Pearson and Iles, 1973; Greene and Spirito, 1979; Zill et al., 2009). Since load transfer and the corresponding proprioceptive impact on leg movement must differ considerably between tethered and freely walking animals (at least if the tether carries or supports the body weight), it is unknown to what extent the results of the connective lesion experiments by Dean (1989) hold for non-tethered walking animals. In contrast to animals in most tethered walking experiments, freely walking animals have to carry their own weight and, therefore, experience load transfer among legs. Moreover, interaction forces between body and substrate differ, not least during yaw rotation of the whole body. Here we investigate how these differences affect temporal and spatial inter-leg coordination in the absence of ipsilateral neural coupling by repeating Dean’s connective lesion experiments in freely walking stick insects. To do so, we recorded protraction/retraction angles of all six thorax-coxa joints in the Indian stick insect *Carausius morosus* (de Sinéty, 1901) after cutting the right connective in the mesothorax or metathorax and compared them with those of animals that had undergone a corresponding sham operation. To ensure natural load transfer among legs, animals were recorded while walking freely across a plane horizontal arena, using marker-based motion capture. We show that stick insects can still walk at similar speed as sham-operated controls, although temporal coordination of legs adjacent to the lesion remains disturbed. Moreover, a detailed comparison of the effects of connective lesions between supported and free walking animals reveals that compensatory adjustments to disrupted neural information transfer concern mainly parameters of spatial coordination among legs, not temporal coordination.

## Materials and Methods

### Animal Preparation

For this study, we used 20 adult, female stick insects of the species *Carausius morosus* (de Sinéty, 1901) from a laboratory colony bred at Bielefeld University. The animals were divided in two cohorts of 10 animals. From each cohort, five animals were assigned to a “treatment group,” whereas the other five were assigned to a “sham group.” Animals of the treatment group underwent an operation in which the right connective was severed between either the pro- and mesothoracic ganglion (Cohort 1) or between the meso- and metathoracic ganglion (Cohort 2). To do so, the animal was fixed on plasticine, ventral side up, and a small incision was made in the cuticula of the meso- or metasternum, using the splinter of razor blade. Then, both connectives were localised by gently moving the tracheae, and the right connective was slightly lifted and cut with fine scissors. Afterward, the incision was closed and sealed with beeswax. The animals of the sham group underwent a corresponding sham operation, in which the same incisions were made to the cuticula, and the connectives were touched gently with tweezers but not cut. Thus, each cohort had its own control group, making sure that any observed changes in locomotion were caused by the treatment, i.e., cutting the connective, and not by the operation itself.

For motion capture, the animals were marked with nine retroreflective markers (Ø 1.5 mm, Prophysics, Zurich, Switzerland). Three of these marked the leg bases and were placed on the dorsal thorax segments between the coxal bases. The other six marked the leg posture, and were placed on the distal, dorsal cuticle of each femur. Markers were fixed to the cuticle with clear nail polish. Marker positions on the body were photographed with a calibrated camera on a stereo lens (Olympus SZ61T with SC30 camera) at an accuracy of 0.1 mm.

### Experimental Procedure

**FIGURE 1:**
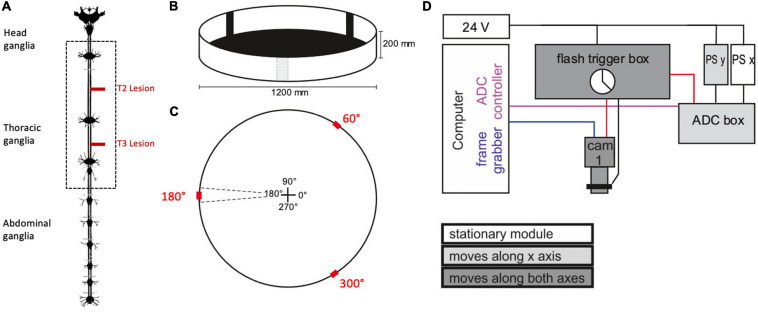
Experimental procedure. **(A)** Nervous system of the stick insect. The dotted box contains the ventral nerve cord ganglia of the thoracic segments (adapted from Marquardt, 1939; the distance between the meso- and meta-thoracic ganglion is approximately 12 mm in adult female *C. morosu*s). For the operation, the right connective was severed either between pro- and mesothoracic ganglion or between the meso- and metathoracic ganglion (T2 and T3 lesion, respectively, red lines). In the sham operations the corresponding connective was touched with a pair of tweezers. **(B)** Animals walked freely in a planar circular arena containing three visual landmarks (black bars) of 10° width. **(C)** Schematic top view of the arena, indicating location and size of the landmarks (red) in relation to the initial walking directions (central cross). **(D)** Schematic wiring diagram of the gantry system for manual two-axis tracking of animals walking within the arena. The camera was mounted to a small sleigh that could be sled along a second, larger sleigh that, in turn, sled along rails on the main frame. The movement of both sleighs was monitored by linear position sensors (PS). The computer ran two programmes that registered the data streams from the camera (blue line: Firewire connection) and the two PS via an analogue-to-digital converter box (ADC box; purple line: USB2 connection. Black lines indicate analogue signals. The clock of the flash trigger box synchronised the cameras, the infrared flashlights, and the ADC box (red lines: TTL connections).

Experiments were carried out in a planar, circular arena (Ø 1,200 mm, height of margin: 200 mm) that was placed below a camera gantry (Figure 1). Before starting the experiment, the animals got a 10 min break for recovery after the operation. Afterwards, they were placed into the arena following a pseudo-random distribution of four cardinal starting directions within the arena (0°, 90°, 180°, 270°). The wall of the arena was illuminated from the outside with a set of eight projectors and a corresponding set of mirrors. As an incentive for walking, three black bars on a white background (width: 10°) were projected onto the arena rim at positions 60°, 180°, and 300°. These bars also served as visual landmarks (Figure 1C).

Prior to each recording the camera view was centred on a point that marked the middle of the arena. Once an animal had been placed onto this point, the video recording was started and the walking animal was followed by shifting the camera on the gantry. The recording was stopped as soon as the animals reached the rim of the arena or stopped walking.

In total, we acquired 69–89 trials per cohort, with each cohort contributing at least 6,200 step cycles to the data set. The total number of trials and step cycles are listed in Supplementary Table 1.

### Data Acquisition

For analysing the pro- and retraction movements, a zoomed-in top view of the walking stick insect was recorded by an infrared-sensitive digital video camera (Basler A602f-2, Basler AG, Ahrensburg, Germany) with a custom-built infrared LED flashlight for illumination and a manual zoom lens (Pentax H6Z810). The camera was mounted to the sled of a custom-made gantry (Item International, Solingen, Germany) with two horizontal movement axes. The camera position above the arena was recorded by two contact-free, linear position sensors (PMS-1-A-1000-K-2410, Megatron, Munich-Putzbrunn, Germany) placed on both axes of the gantry. The camera shutter, flashlight and camera position record were synchronised to via TTL pulses generated by a custom-built flash trigger box (Michael Dübbert, Electronics workshop of Zoological Institute, University of Cologne; Figure 1D). The experimenter could manually move the camera along the two gantry axes, while observing the live image on a computer screen.

Videos were recorded with 50 frames per second at resolution of 480 × 640 pixels, and captured via Firewire (IEEE 1394) using a custom-written frame grabber software (Sven Hellbach and Peter Iseringhausen, Bielefeld University) that generated videos in AVI format, along with a separate text file with time stamps for individual frames. Camera position was recorded via USB using an analogue-to-digital converter (Data Translation DT9802, Data Translation GmbH, Bietigheim-Bissingen, Germany) that also registered a binary camera exposure signal for later alignment of video and camera position data.

### Data Analysis

Data analysis was done in MATLAB (The Mathworks, Natick, United States) using custom-written scripts and graphical user interfaces (GUIs). In a first step, the position records from the gantry system and the time stamps of the video recording software were aligned, yielding the 2D position time course of the camera. In a second step, the recorded videos were processed, yielding image positions of the nine markers for each video frame. To do so, markers were assigned and labelled manually in the first frame and then tracked semi-automatically using threshold-based clustering of marker pixels and a nearest-neighbour tracking algorithm. In a third step, the gantry position data, time stamps, and extracted marker coordinates were combined with calibration data for the camera projection and arena properties in separate files per trial.

These combined data files allowed calculation of both external, arena-centred information such as body orientation and velocity, and local, body-centred information about leg coordination. For the latter, positions were expressed relative to a “root marker” (in our case, the marker on the posterior metathorax) and aligned with the body axis. The resulting body-centred marker trajectories were used to calculate the time courses of protraction/retraction angles of all thorax-coxa joints. Protraction/retraction of a leg was defined as the angle between the line connecting the femoral and thoracic marker and the line perpendicular to the body axis. As a result, an angle of zero indicates that the femur was orthogonal to the body axis, and a positive angle indicates that the femur pointed forward. Extraction of local maxima and minima from protraction/retraction time courses yielded the times of movement reversals at the thorax-coxa joints. These served as estimates of the lift-off and touch-down events and thus, the onset/offset of stance and swing phases. Note that this definition of swing and stance phases is common in the literature (e.g., Wendler, 1964; Dean, 1989) but neglects small phase shifts between the protraction/retraction cycle of the thorax-coxa joint and the actual onset/offset of ground contact (e.g., Theunissen et al., 2015, see their Figure 9). Also, all positional step cycle parameters like step length, anterior and posterior extreme positions correspond to angles and will be given in degrees.

Body position and orientation within the arena were calculated by combining the camera position relative to the gantry and marker positions within each video frame. Forward and sideward translational velocities [mm/s] and yaw rotational velocity [deg./s] were calculated from the shift and rotation of the animal between subsequent frames and smoothed by use of a sliding median filter with a window of 60 ms (3 frames). For further information about data analysis and sample data, see Supplementary Material.

Because each one of the five animals per cohort contributed a lot of steps, the statistical analysis had to take into account the large but unbalanced samples per animal, for *n* = 5 independent samples per cohort. This was done in a two-step procedure by first re-sampling balanced pooled distributions with the original total sample size, and then bootstrapping the median and its 95 and 99% confidence intervals from 10,000 balanced samples. Statistical significance of pairwise comparisons was concluded whenever the 95% confidence intervals (95% CI) did not overlap (*p* < 0.05). The corresponding pairwise effect sizes were calculated as differences between cohort medians, divided by their mean 95% CI. Circular statistics on phase differences between step cycles were calculated on per-animal means, using the MATLAB toolbox CircStat (Berens, 2009).

## Results

### General Observations

**FIGURE 2:**
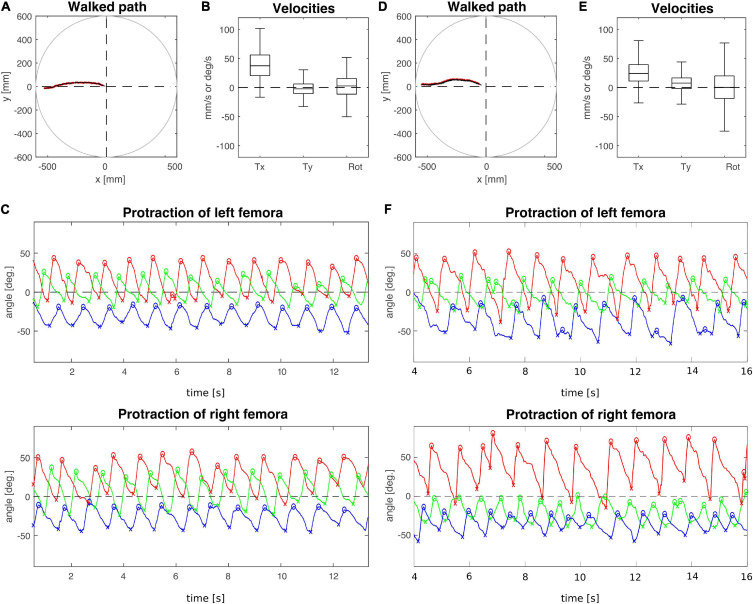
Representative trials of animals that had undergone an operation at the pro-to-mesothorax connective. **(A–C)** T2 sham operation; **(D–F)** T2 connective lesion. **(A,D)** Walked path of the animal in the circular arena. The animal was placed in the centre of the arena facing toward one of four starting directions (here: 180°, see also Figure 1C) and walked toward a visual landmark on the arena wall in that same direction. **(B,E)** Fluctuation of forward (T_*x*_) and sideward (T_*y*_) translational velocities (in mm/s) as well as the rotational velocity about the yaw axis (in deg/s). **(C,F)** Time courses of protraction/retraction angles of the animals’ front (red), middle (green) and hind legs (blue). Zero degrees (black dashed lines) corresponds to a leg posture orthogonal to the body axis. The anterior and posterior extreme positions are marked by circles and crosses. Note that the time course of the trial shown in **(F)** was truncated to the same time window as the trial shown in **(C)**. The complete trial is shown in Supplementary Figure 2.

To analyse the effect of connective lesions on walking behaviour, we will first provide a general overview of the walking parameters of representative, single trials and later quantify the effects on both temporal and spatial parameters of inter-leg coordination across the different cohorts. Figure 2 compares trials from animals with a lesion (T2 lesion) or sham operation (T2 sham) at the pro-to-mesothorax connective. Despite the fact that both animals walked a similar path, several aspects differed between the sham-operated and lesioned animal. First, the lesioned animal was slower and showed a leftward bias in sideward velocity (Figures 2B,E). Furthermore, the local minima and maxima of the protraction angles revealed pronounced shifts of several extreme positions and/or working ranges of the different legs (Figures 2C,F). Compared to the sham-operated animal, the left front and hind legs (intact side) of the lesioned animal took bigger steps by shifting their posterior extreme positions (PEP) to the rear. Also, the left hind leg extended the stance phase such that it tended to lift off later than the front leg (compare blue and red crosses at local minima in Figure 2F). On the right side (treatment side), the anterior extreme position (AEP) of the front leg is strongly shifted forward, resulting in much larger steps. Moreover, the working range of the right middle leg decreased and shifted rearwards.

**FIGURE 3:**
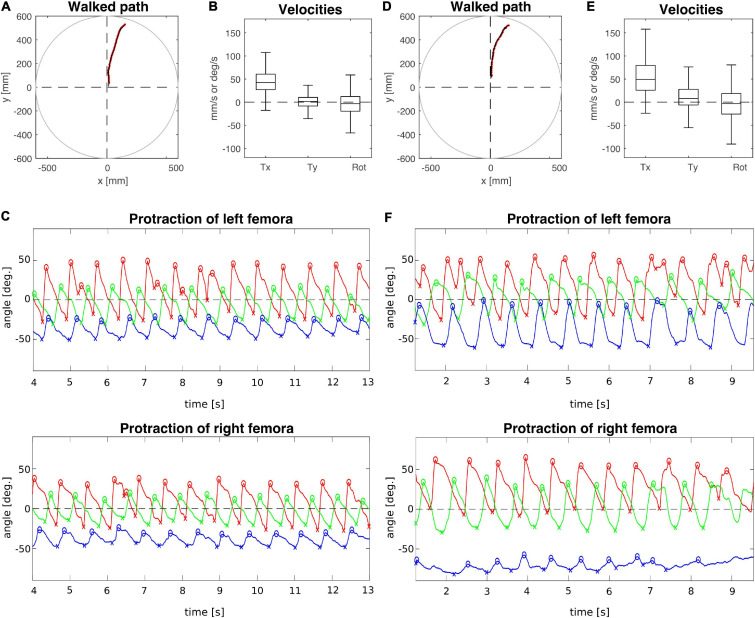
Representative trials of two animals that had undergone an operation at the meso-to-metathorax connective. **(A–C)** T3 sham operation; **(D–F)** T3 connective lesion. Same graphics details as in Figures 2A,D: Walked path of the animal in the circular arena. Here, the animals started to walk in cardinal direction 90° (see Figure 1C) and turned toward the border of a visual landmark on the arena rim at 60 deg. **(B,E)** Fluctuation of forward (T_*x*_) and sideward (T_*y*_) translational velocities (in mm/s) as well as the rotational velocity about the yaw axis (in deg/s). **(C,F)** Time courses of protraction/retraction angles of the animals’ front (red), middle (green) and hind legs (blue). Zero degrees (black dashed lines) corresponds to a leg posture orthogonal to the body axis. The anterior and posterior extreme positions are marked by circles and crosses. Note that the time course of the trial shown in **(C)** was truncated to the same time window as the trial shown in **(F)**. The complete trial is shown in Supplementary Figure 3.

In contrast, the animal with a lesioned right meso-to-metathorax connective shown in Figure 3 (T3 lesion) was still capable of walking at a similar forward velocity as the sham-operated animal (T3 sham), but also revealed a bias in sideward translational velocity to the left (Figures 3B,E). The protraction angles of the legs show that the hind leg of the (right) treatment side executed only very small and seemingly uncoordinated protraction movements. Also, its working range was strongly shifted rearwards. At the same time, the opposite (intact side) hind leg showed a strongly increased step length, caused by a forward shift of the AEP and a rearward shift of the PEP. Also, this leg stayed retracted at nearly the same angle for some time before lift-off. This may indicate a further rearward shift of the foot by extension of the femur-tibia and/or depression of the coxa-trochanter joints which was not monitored. Compared to the sham-operated animal, the working ranges of the right front and middle legs of the lesioned animal were enlarged and shifted forward. The opposite front and middle legs showed little to no change in their protraction/retraction time courses.

Taken together, these representative trials show that a number of effects were induced on the treatment side, but several adjustments concerned the opposite, intact body side, too. In the next sections, we examine the consistency of these lesion-induced differences across entire cohorts.

### Effects on Velocity and Step Cycle Parameters

**FIGURE 4:**
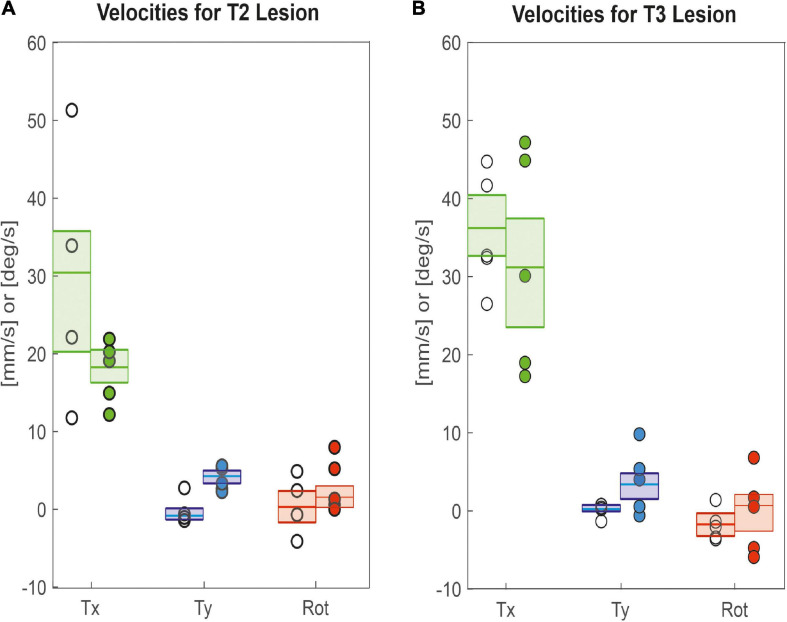
Lesion effects on translational and rotational velocities. Tx: Forward translational velocity (green); Ty: Sideward translational velocity, with positive values indicating shifts to the left (blue); Rot: Rotational velocity, with positive values indicating ccw rotation (red). **(A)** Pro-to-mesothorax connective; **(B)** meso-to-metathorax connective. Symbols show median velocity per animal (lesion: filled circles; sham operation: open circles). Boxes comprise all trials of the cohort and show the median velocities and the bootstrapped 95% CI.

As animals were walking freely on a horizontal plane, we could determine all three degrees of freedom of motion in the plane and assess lesion-induced effects on both translational velocities (Tx: forward; Ty: sideward) and rotational velocity about the yaw axis. Figure 4A shows that animals with a lesioned pro-to-mesothorax connective walked with significantly increased sideward velocity (Ty_*sham*_ = −0.8 mm/s, Ty_*lesion*_ = 4.2 mm/s, *p* < 0.05) and tended to walk slightly slower than sham-operated animals, but the latter difference was not statistically significant (Tx_*sham*_ = 30.4 mm/s, Tx_*lesion*_ = 18.2 mm/s, n.s.). Similarly, animals with a meso-to-metathorax connective lesion (Figure 4B) walked at a similar forward velocity as sham-operated animals (Tx_*sham*_ = 36.2 mm/s, Tx_*lesion*_ = 31.1 mm/s, n. s.). As for the other lesion, these animals walked at a significantly increased sideward velocity (Ty_*sham*_ = 0.2 mm/s, Ty_*lesion*_ = 3.3 mm/s, *p* < 0.05). Neither lesion resulted in a change of median yaw rotation.

**FIGURE 5:**
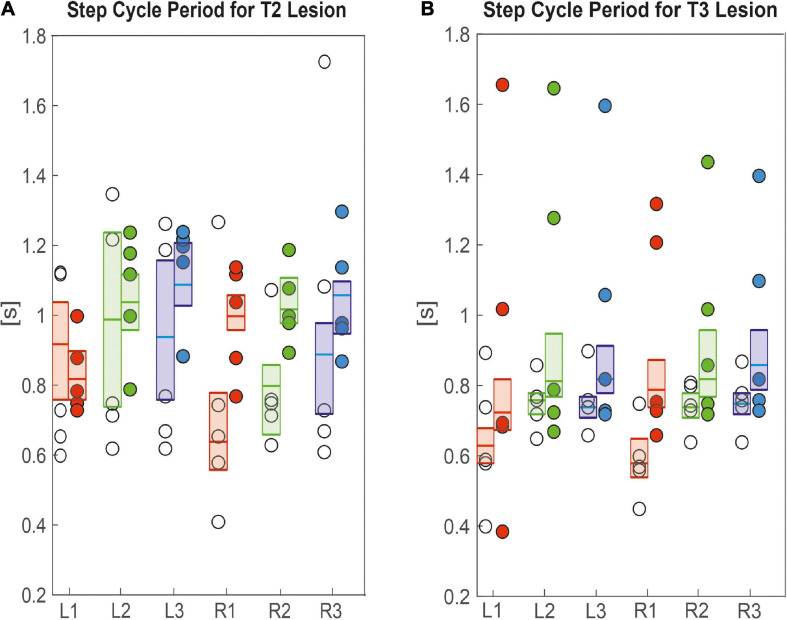
Effects on step cycle period. **(A)** Pro-to-mesothorax connective; **(B)** meso-to-metathorax connective. Symbols show the median step cycle period per animal after lesion (filled circles) or a sham operation (open circles). Boxes comprise all trials of the cohort and show the median and the bootstrapped 95% CI.

Since these differences in velocity can be due to changes in the step length and step cycle period, we took a closer look at these parameters. Figure 5A shows that R1 and R2 legs of animals with a lesion of the right pro-to-mesothorax connective, i.e., the legs immediately anterior and posterior to the lesion, had significantly longer step cycle periods than sham-operated animals (R1_*s*__*ham*_ = 0.6 s, R1_*l*__*esion*_ = 0.9 s, *p* < 0.01; R2_*s*__*ham*_ = 0.7 s, R2_*l*__*esion*_ = 1.0 s, *p* < 0.01). The step cycle period of all other legs showed no statistically significant differences. Following a lesion of the meso-to-metathorax connective lesion (Figure 5B), both hind legs (L3, R3) as well as the right front leg (R1) showed a significantly increased step cycle period after the lesion (L3_*s*__*ham*_ = 0.7 s, L3_*l*__*esion*_ = 0.8 s, *p* < 0.01; R3_*s*__*ham*_ = 0.7 s, R3_*l*__*esion*_ = 0.8 s, *p* < 0.05; R1_*s*__*ham*_ = 0.5 s, R1_*l*__*esion*_ = 0.7 s, *p* < 0.05). Generally, lesioned animals showed a large variance in step cycle period (Figure 5B). For effect sizes see Table 1 (T2 lesion) and Table 2 (T3 lesion).

**TABLE 1:** Summary of the changes in step parameters induced by a lesion of the pro-to-mesothorax connective.

**T2 lesion**	**Lesion**									**Sham**		
	**Local changes**						**Bilateral asymmetry**			**Bilateral asymmetry**		
	**L1**	**L2**	**L3**	**R1**	**R2**	**R3**	**FL**	**ML**	**HL**	**FL**	**ML**	**HL**
AEP	↓ 1.57	↑↑ 3.84	↑↑ 1.43	↑↑ 3.29	↓↓ 6.67	↓↓ 1.85	↑↑ 5.48	↓↓ 9.94	↓↓ 3.89		↑ 1.45	
PEP	↓ 1.42	↑↑ 1.83	↓ 1.14	↑↑ 2.6	↓↓ 3.05	↓↓ 1.73	↑↑ 3.81	↓↓ 3.75	↑ 1.67		↑↑ 1.61	
LENGTH		↑ 0.98	↑↑ 3.02	↑ 1.33	↓↓ 2.79		↑↑ 1.61	↓↓ 4.4	↓↓ 3.1			
DURATION				↑↑ 2.25	↑↑ 1.33		↑ 1.5			↓ 1.12		

**The statistical significance and direction of the effect (arrows) as well as the effect size (numbers) are shown for local changes in single leg step cycles and bilateral leg pairs of lesioned and sham-operated animals. Arrows indicate the direction and significance level (one arrow: p < 0.05; two arrows: p < 0.01). Effect sizes more than twice the 95% CI are coloured in dark red, effect sizes larger than four times the 95% CI are coloured in bright red. Grey shading of local changes columns indicates the location of the lesion.**

**TABLE 2:** Summary of the changes in step parameters induced by a lesion of the meso-to-metathorax connective.

**T3 lesion**	**Lesion**									**Sham**		
	**Local changes**						**Bilateral asymmetry**			**Bilateral asymmetry**		
	**L1**	**L2**	**L3**	**R1**	**R2**	**R3**	**FL**	**ML**	**HL**	**FL**	**ML**	**HL**
AEP	↑↑ 1.25	↑↑ 3.41	↑ 1.18	↑ 1.61	↑↑ 6.37	↓↓ 5.15			↓↓ 4.11			↑↑ 1.53
PEP		↑ 1.19	↓↓ 2.46			↓↓ 5.12			↓ 1.32		↑ 2.17	↑ 1.16
LENGTH	↑ 1.38	↑↑ 3.86	↑↑ 3.29		↑↑ 2.41	↓↓ 2.76	↑↑ 1.67		↓↓ 4.03	↓↓ 1.14		
DURATION			↑ 0.8	↑↑ 1.68		↑ 0.95						

**The statistical significance and direction of the effect (arrows) as well as the effect size (numbers) are shown for local changes in single leg step cycles and bilateral leg pairs of lesioned and sham-operated animals. Same details as in Table 1.**

**FIGURE 6:**
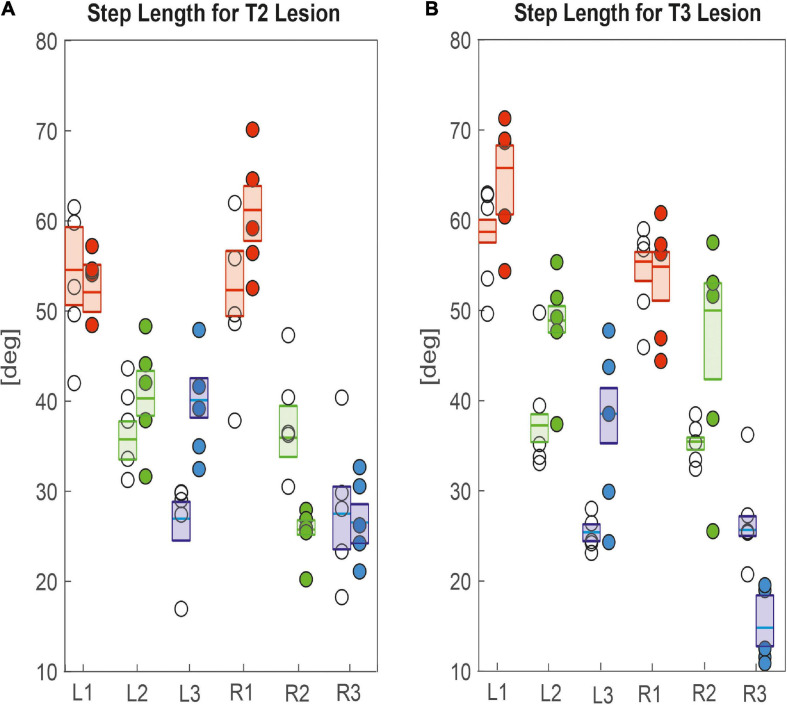
Effects on step length. **(A)** Pro-to-mesothorax connective; **(B)** meso-to-metathorax connective. Symbols show median step length per animal with connective lesion (filled circles) or sham operation (open circles). Boxes comprise all trials of the cohort and show the median step length and the bootstrapped 95% CI.

Other than step cycle period, step length was generally affected more, both in terms of effect size and in number of legs (Figure 6), corroborating the effects seen in the single trials shown in Figures 2, 3. After cutting the right pro-to-mesothorax connective, the leg posterior to the lesion took smaller steps (R2_*s*__*ham*_ = 35.9 deg., R2_*l*__*esion*_ = 25.7 deg., *p* < 0.01) while the leg anterior to the lesion took larger steps (R1_*s*__*ham*_ = 52.3 deg., R1_*l*__*esion*_ = 61.2 deg., *p* < 0.05). Furthermore, the contralateral hind and middle legs showed significantly increased step lengths (L2_*s*__*ham*_ = 35.7 deg., L2_*l*__*esion*_ = 40.3 deg., *p* < 0.01; L3_*s*__*ham*_ = 26.9 deg., L3_*l*__*esion*_ = 40.1 deg., *p* < 0.01). Similarly, animals with a lesioned meso-to-metathorax connective showed altered step lengths of the legs anterior and posterior to the lesion (Figure 6B). The right hind leg took significantly smaller steps (R3_*s*__*ham*_ = 25.6 deg., R3_*l*__*esion*_ = 14.8 deg., *p* < 0.01) while the middle leg took larger steps (R2_*s*__*ham*_ = 35.4 deg.; R3_*l*__*esion*_ = 49.9 deg., *p* < 0.01). Moreover, all three contralateral legs took longer steps compared to sham-operated animals (for *p*-values and effect sizes see Table 2).

Taken together, the similar forward velocity with and without lesion of the pro-to-mesothorax connective was mirrored by fairly consistent step cycle periods in four of six legs, whereas the changes in step length overall larger and left only two leg unaffected (Table 1). Similarly, our finding that animals with a cut meso-to-metathorax connective could walk equally fast as sham-operated animals was mirrored by an overall weaker change in step cycle period (concerning three legs) and overall stronger and more wide-spread change in step length (concerning five legs).

### Spatial Coordination

**FIGURE 7:**
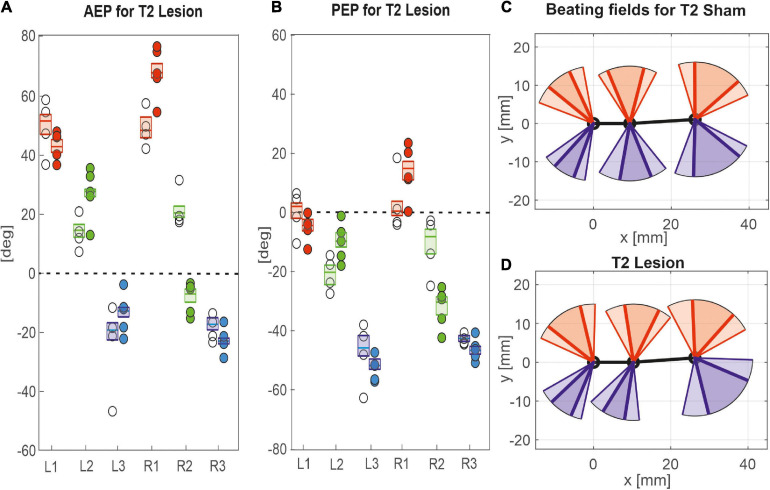
Effects of the T2 lesion on anterior and posterior extreme positions. AEPs **(A)** and (PEPs **(B)** of all legs following an operation at the pro-to-mesothorax connective. Symbols show median extreme positions per animal with a cut connective (filled circles) or with a sham operation (open circles). Boxes show the distributions for all trials per cohort with the median extreme positions and 95% CI. Zero degrees (black dashed lines) corresponds to a leg posture orthogonal to the body axis. **(C,D)** Beating fields show both the size and boundaries of the working range of each leg with a sham operation in the mesothorax (**C**, top) or with a cut pro-to-mesothorax connective (**D**, bottom). Schematic top views indicate the median femoral postures at the AEP and PEP for the left (red) and right legs (blue) in relation to the body axis (black). Transparent areas show the corresponding 5% percentiles of the PEP and 95% percentiles of the AEP.

**FIGURE 8:**
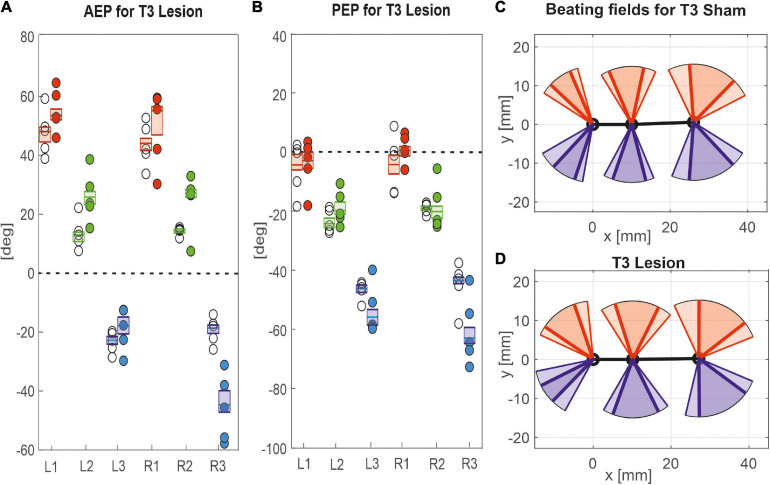
Effects of the T3 lesion on anterior and posterior extreme positions. AEPs **(A)** and PEPS **(B)** of all legs following an operation at the meso-to-metathorax connective. Symbols show median extreme positions per animal with a cut connective (filled circles) or with a sham operation (open circles). Boxes show the distributions of all trials per cohort with the median extreme positions and 95% CI. **(C,D)** Beating fields show both the size and boundaries of the working range of each leg with a sham operation in the metathorax (**C**, top) or with a cut meso-to-metathorax connective (**D**, bottom). Same graph details as in Figure 7.

Given the leg-specific changes in step length, we further examined how these changes in step length related to forward or rearward shifts of the actual touch-down and lift-off positions. To this end Figures 7, 8 show the protraction/retraction angles at the onset of swing or stance, which we interpret as equivalents of the anterior (AEP) and posterior extreme positions (PEP) of all legs. Figure 7 shows the effect a cut pro-to-mesothorax connective. All six legs significantly shifted both AEP and PEP, though with strongly different effect sizes (Table 1). The strongest effect was observed for the median AEP (Figure 7A) and PEP (Figure 7B) of the right middle leg, both of which strongly shifted to the rear compared to sham-operated animals (R2: AEP_*lesion*_ = −6.9 deg., *p* < 0.01; PEP_*lesion*_ = −30.3 deg., *p* < 0.01). Also, the working range of the right front leg shifted anteriorly by forward shifts of both the AEP and PEP (R1: AEP_*lesion*_ = 67.9 deg., *p* < 0.01; PEP_*lesion*_ = 14.8 deg., *p* < 0.01). Furthermore, the AEP of the contralateral hind leg also shifted forward (L3: *p* < 0.05), thus leading to the increase of step length observed in Figure 6A. Finally, both AEP and PEP of the contralateral middle leg shifted anteriorly (L2: AEP_*lesion*_ = 27.7 deg., *p* < 0.01; PEP_*lesion*_ = −9.7 deg., *p* < 0.01), resulting in a forward shift of the working range with a small change in step length only (compare beating fields in Figures 7C,D).

Similar to the results described above, a lesion of the meso-to-metathorax connective affected the touch-down and lift-off locations of most legs, again with very different effect sizes (Table 2). Again, effects were strongest anterior and posterior to the lesion (Figure 8). The median AEP and PEP of the right hind leg both shifted to the rear (R3: AEP_*lesion*_ = −44.7 deg., *p* < 0.01; PEP_*lesion*_ = −62.9 deg., *p* < 0.01), whereas the median AEP of the right middle leg strongly shifted forward (R2: AEP_*lesion*_ = 27.1 deg., *p* < 0.01) with no change of the PEP. As a consequence, the narrowed working range of the right hind leg revealed a strong rearward shift, whereas the broadened working range of the right middle leg shifted only little (compare Figures 8C,D). Of the legs contralateral to the lesion, all legs showed a significant anterior shift of the AEP, while the effect on the PEP differed among legs: Whereas the PEP of the left middle leg shifted forward (i.e., in the same direction as the AEP), it shifted rearward in case of the left hind leg (i.e., in the opposite direction of the AEP) resulting in a strong increase in step length (compare beating fields of L3 in Figures 8C,D).

Taken together, these results show leg-specific, local shifts of both AEP and PEP, with particularly strong effects on the legs anterior and posterior to the lesion. The fact that all legs of the intact (left) body side also underwent significant changes after lesion highlights the complex interplay of local adjustments in spatial coordination, potentially caused by direct effects of the lesion as well as by local compensatory effects on both body sides.

### Temporal Coordination

**FIGURE 9:**
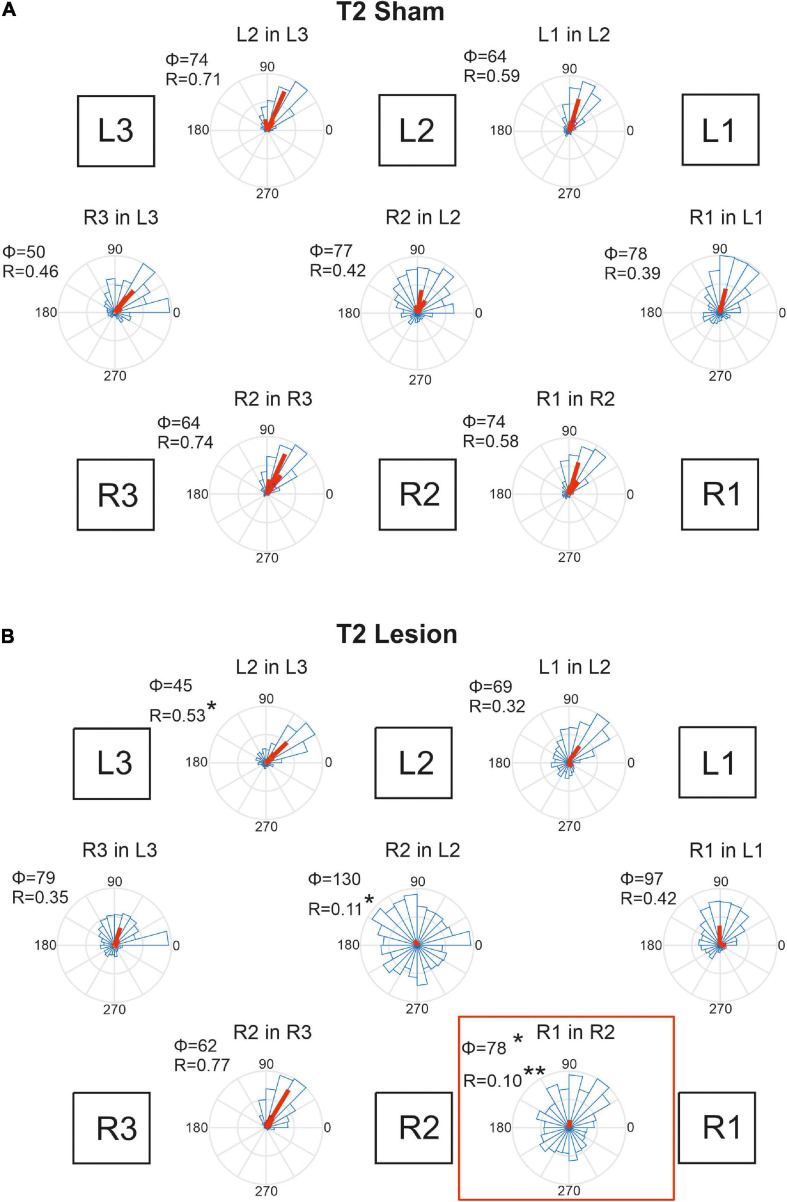
Temporal coordination of step cycles after an operation in the mesothorax. **(A)** Sham-operated animals. **(B)** Animals with a lesion of the right pro-to-mesothorax connective (T2 lesion). Square boxes labelled L1–L3 and R1–R3 show the arrangement of the six legs. For each leg pair labelled “Leg1 in Leg 2”, rose plots show pooled distribution (blue) and per-animal mean phase shifts Φ (red) of the onset of swing by Leg 1 (receiver leg) in relation to the step cycle of (sender) Leg 2. Accordingly, Φ = 0 indicates that L1 lifted off at the same time as L2 touched down. Circular histograms comprise all steps per cohort in 15 deg. bins. Statistics were calculated on per-animal mean phase vectors, with Φ and R giving the corresponding angle and length of that vector, respectively. Significance levels, *p < 0.05; **p < 0.01.

**FIGURE 10:**
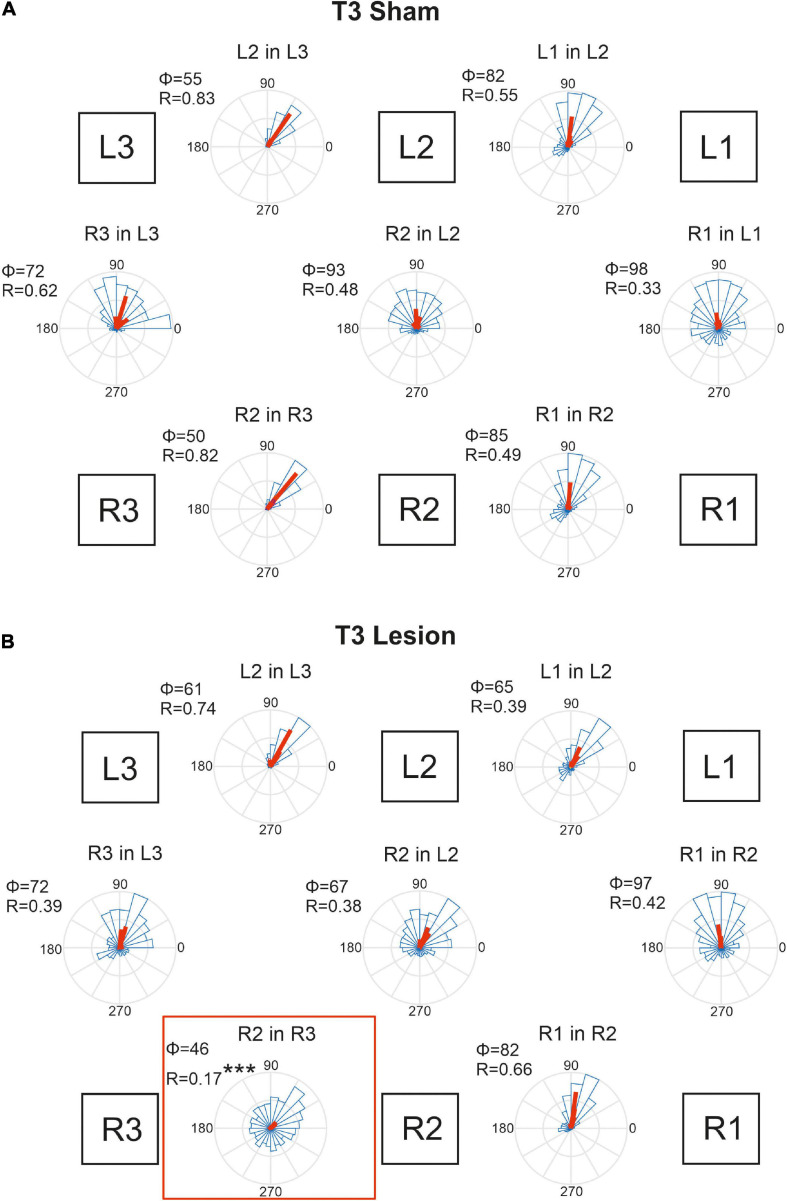
Temporal coordination of step cycles after an operation in the metathorax. **(A)** Sham-operated animals. **(B)** Animals with a lesion of the right meso-to-metathorax connective (T3 lesion). Same graph details as for Figure 9. Significance level ***p < 0.001.

Given the fact that the observed spatial adjustments were not equal across all legs, despite the fact that animals with connective lesions could walk along the same paths as sham-operated animals (Figures 1, 2), and even at a similar speed (Figure 2), a major question was to find out which changes in temporal coordination kept walking sufficiently coherent. In particular, we were interested in potential changes in pairwise coupling of ipsilateral leg pairs according to Cruse’s rule 2, i.e., the rule that a receiver leg commences a swing movement shortly after touch-down of its (posterior) neighbouring sender leg. Therefore, for each ipsilateral leg pair we registered the onset of a swing phase of the anterior (receiver) leg and related it to the step cycle of its posterior neighbour (sender leg). The same was done for contralateral leg pairs, expressing the phase of the onset of swing on the operated (right) body side relative to the step cycles of their neighbours on the intact (left) body side. In all cases, the reference step cycle of the sender leg was defined as the interval between the two subsequent anterior extreme positions, i.e., touch-down events. This choice allowed us to interpret the phase shift in the context of Cruse’s coordination rule 2, but also in relation to the unloading event due to load transfer from sender to receiver legs. The corresponding rose plots of Figures 9, 10 show the mean phase shift per animal and the dispersion of the pooled distribution, the latter being a measure of coupling strength between leg pairs. All circular statistics reported below were calculated on per-animal means.

Figure 9A shows that the phase shifts were very similar for all leg pairs in case of the T2 sham operation. Typically, the receiver leg lifted off in the late first quarter of the step cycle of the sender leg, with mean phase angles ranging between 50 and 78 deg. This coherent pattern of coordination was disrupted after the lesion of the right pro-to-mesothorax connective (Figure 9B, T2 Lesion). After lesion, the right front leg started its swing phase without obvious coupling to the step cycle of the right middle leg. Although the mean phase shift changed only little compared to that of the sham-operated cohort (R1 in R2: φ_*sham*_ = 74; φ_*lesion*_ = 78, *p* < 0.05, Watson-Williams test), we observed a strong increase in dispersion of phase and, as a result, decreased coupling strength (R1 in R2: r_*sham*_ = 0.58, r_*lesion*_ = 0.10, *p* < 0.01, Kuiper test). Both effects were statistically significant. Not only the temporal coordination of the ipsilateral leg pair adjacent to the lesion was affected, but also the contralateral coupling between the left and right middle legs: The right middle leg tended to start its swing movement later in the step cycle of the left middle leg (R2 in L2: φ_*sham*_ = 77, φ_*lesion*_ = 130; n.s.), but the dispersion was similarly increased as for the ipsilateral leg pair (R2 in L2: r_*sham*_ = 0.417; r_*lesion*_ = 0.11, *p* < 0.05). Owing to the variation of per-animal mean phase, only the effect on dispersion was statistically significant. A further effect concerned the ipsilateral coupling of the hind and middle legs of the (left) intact body side, that showed a reduced mean phase angle (L2 in L3: φ_*sham*_ = 74, φ_*lesion*_ = 45, *p* < 0.05). Related to these changes we observed a tendency for increased phase angles between contralateral front and hind leg pairs. Whereas the mean phase shift of these contralateral pairs was very similar to that of the left ipsilateral leg pairs in sham-operated animals (compare L2-in-L3 to R3-in-L3, and L1-in-L2 to R1-in-L1 in Figure 9A), there is a consistent increase of phase angle for all legs of the operated side with reference to their contralateral neighbours on the intact body side. Given the fact that the coordination of R2-in-R3 remained unaffected by the lesion, despite the weaker coupling of R2 and either L2 and R1, we suggest that the changes in contralateral coordination and ipsilateral coordination on the intact side are secondary effects. They could be a consequence of maintaining coherence among the six legs, with the primary lesion effects concerning R1-in-R2 and R2-in-L2.

Animals that had undergone a sham operation in the metathorax (Figure 10A) showed similar temporal coordination pattern as did the cohort with a sham operation in the mesothorax (Figure 9A). In all contralateral and ipsilateral leg pairs the receiver leg commenced swing with a phase lag between 50 and 98 deg, relative to touch-down of the sender leg. A small but notable difference between the T2 sham (Figure 9A) and T3 sham cohorts (Figure 10A) concerned the slightly larger mean phase shifts of the front legs (R1-in-R2 and L1-in-L2) compared to that of the middle legs (R2-in-R3 and L2-in-L3).

Figure 10B shows that the T3 lesion of the right meso-to-metathorax connective had a similar effect on the adjacent ipsilateral leg pair as described above for the T2 lesion cohort. After the lesion, the lift-off of the right middle leg was almost randomly distributed in the step cycle of the posterior right hind leg. Whereas the mean phase shift remained similar as in sham-operated animals, angular dispersion was very large, i.e., coupling strength was weak (R2 in R3: φ_*sham*_ = 50, φ_*lesion*_ = 47, n.s.; r_*sham*_ = 0.82; r_*lesion*_ = 0.17, *p* < 0.001). The phase relation between the contralateral hind legs, however, did not change after the lesion (φ_*sham*_ = 72, φ_*lesion*_ = 72, n.s.) and coupling strength decreased only slightly and non-significantly (r_*sham*_ = 0.62, r_*lesion*_ = 0.39, n.s.). A secondary effect involving the intact (left) legs and contralateral coupling was weaker than described for the T2 lesion above. As yet, we observed a slightly decreased phase angle for the contralateral pair of middle legs in Figure 10B (R2 in L2), but this change was statistically non-significant.

Taken together, these results show that connective lesions affected the temporal coordination of leg pairs only locally, i.e., not consistently among legs. After both types of lesion, the main effect concerned the leg posterior to the lesion, indicating that ipsilateral coupling is strongly affected by disruption of neuronal information transfer from anterior to posterior legs.

## Discussion

### Coordination Rules, Load Transfer, and Motor Flexibility

Recent findings in cockroaches and stick insects revealed that mechanical transfer among ipsilateral legs can be sensed by campaniform sensilla at the base of the insect leg, and may contribute to maintain temporal coordination (cockroach: Zill et al., 2009; stick insect: Dallmann et al., 2017) without involving intersegmental neurons. On the other hand, several studies have investigated the effect of thoracic connective lesions on inter-leg coordination (e.g., *Blatta*: Hughes, 1957; *Periplaneta*: Pearson and Iles, 1973; Greene and Spirito, 1979; *Carausius*: Dean, 1989), and all of them concluded that neural information transfer through thoracic connectives is important for temporal coordination of the adjacent, ipsilateral pair of legs. However, all analyses in the mentioned studies dealt with inter-leg coordination in tethered animals [some, with anecdotal remarks on free walking), and except for Greene and Spirito (1979); for method see Spirito and Mushrush, 1979] the animals were supported, thus altering the nature and reducing the magnitude of sensory feedback about load. Moreover, only the study of Dean (1989) has analysed the effect of connective lesions on spatial coordination among legs. Owing to the significance of spatial coordination for the resulting load distribution among legs and, therefore, for mechanical load transfer between legs (for examples in biology and biomimetics see Dallmann et al., 2017; Dürr et al., 2019, respectively), the aim of the present study was to assess the potential of mechanical load transfer in insect walking without neural coupling of ipsilateral leg pairs. To this end, we analysed both spatial and temporal inter-leg coordination of unrestrained walking stick insects with and without severed thoracic connectives.

A conceptual framework for behavioural analysis of inter-leg coordination has been established by Cruse and coworkers, who derived a set of inter-leg coordination rules (Cruse, 1990) that has set the stage for detailed experimental analysis (temporal coordination: e.g., Kindermann, 2002; Dürr, 2005; spatial coordination: e.g., Schumm and Cruse, 2006; Theunissen et al., 2014) and modelling (e.g., Espenschied et al., 1996; Cruse et al., 1998; Schilling and Cruse, 2020) of hexapedal locomotion. Cruse’s rules describe interactions among adjacent leg controllers that depend on their current state (being either the thrust-generating stance phase or the re-positioning swing phase) and local mechanosensory information about posture, ground contact and/or load. Last not least, because of the different coupling strengths between different leg pairs (Dürr, 2005; Grabowska et al., 2012) and context-dependent modulation of coupling strengths (Dürr, 2005), Cruse’s concept of how gaits and gait transitions emerge through distributed interaction of pairwise, mutually coupled leg controllers offers a valuable framework for understanding motor flexibility in general (for review, see Dürr et al., 2018).

With regard to load transfer among legs, Cruse’s rules 1 and 2 are of particular interest, both of which operate from a posterior “sender leg” to its anterior “receiver leg.” Rule 1 states that during swing phase the sender leg inhibits the start of a swing movement in the adjacent receiver leg. Rule 2 regulates the onset of a swing movement of the receiver leg depending on the onset of stance in the sender leg. In both cases, the crucial timing event is the touch-down of the sender leg that, by taking on load, induces mechanical load transfer from the receiver leg to the sender leg. To test whether Cruse’s rules 1 and 2 require neural information transfer, Dean (1989) tested ipsilateral coupling of leg pairs after cutting thoracic connectives. His results showed that the coordination of the legs immediately adjacent to the lesion was hampered significantly, leading to the conclusion that Cruse’s rules 1 and 2 should be implemented by some sort of anteriorly directed neural information travelling through the ipsilateral connective. Although Dean’s conclusions are perfectly valid for an experimental situation without mechanical load transfer, recent insights into the mechanisms underlying mechanical load transfer in insects (Zill et al., 2009; Dallmann et al., 2017) call for a re-investigation under naturalistic load distribution. To this end, we measured temporal and spatial coordination parameters of visually guided but mechanically unrestrained walking stick insects (*Carausius morosus*) in a planar arena after severing one thoracic connective.

To account for Hughes’ warning that “in any experiment involving operations such as these it is often difficult to distinguish the effects produced by the specific operation from those resulting from the general injury” (Hughes, 1957, p. 323) we designed the study to compare lesioned animals with animals that underwent a corresponding sham operation (other than Dean, 1989, who conducted a “before-after” study). A potential limitation of our experimental design concerns our decision to opt for a relatively small number of individuals (*N* = 5 per cohort) with the benefit of having many step cycles per animal and reliable estimates of per-animal means. To improve comparability with Dean’s results, we did not differentiate between distinct classes of step types (Theunissen and Dürr, 2013). Although short steps are known to be relatively infrequent in planar walking, it is worth to bear in mind that neglecting them would have mainly concerned observations on front legs, where short steps are most frequent. Finally, to account for the fact that insects are known to adjust to connective lesions (Greene and Spirito, 1979) or genetic manipulation of mechanoreceptive input (Isakov et al., 2016) over time, we focussed on immediate effects of the lesion only (as opposed to long-term effects that, in cockroaches, establish over a period of about 3 weeks; Greene and Spirito, 1979).

As a further methodological note, it is useful to bear in mind the differences in data acquisition by Dean (1989) and us: Dean’s optical recording system measured the tangent of the protraction/retraction angle, rather the leg angle itself, as reported here. This makes it difficult to compare effect sizes, as both angle and dispersion estimates by Dean (1989) were subject to a non-linear transformation.

### Under Load, Connective Lesions Affect Spatial Coordination More Widely and Strongly Than Temporal Coordination

In a qualitative description of the effects of a T2 lesion in free walking stick insects, Dean (1989) noted that “the ipsilateral middle leg usually remained in a posterior position, where it was dragged over the ground. Because the ipsilateral front and rear legs together provide sufficient support for the animal during their common stance, the middle leg was sometimes able to make long, slow swing movements” (Dean, 1989, p. 116). This turned out quite differently in our experiments, as the middle leg posterior to the lesion regularly engaged in rhythmic movements, albeit with an altered working range (see Figure 2).

Qualitatively, Dean’s observation that in tethered animals a connective lesion strongly affected the legs immediately adjacent to the lesion, with multiple other, often minor effects, was the same in our free walking animals. However, the results differed quite strongly when comparing some details, even for the adjacent leg pair. For example, Dean (1989) found after a T2 lesion that “the mean AEP and PEP of the ipsilateral […] middle leg, showed little change but their standard deviations increased” (p. 116). Comparing his Table 4 with our Table 1 reveals that in free walking, the effects on that middle leg were among the strongest found in free walking animals (the rearward shift of the AEP was more than six times the 95% CI). In case of the PEP, shifts even were of opposite sign: We found a strong rearward shift, Dean found a slight forward shift. At the same time, we did not find a consistent increase of the 95% CI, which is in contradiction with Dean’s observation of increased spread after lesion. Related to these differences, Dean (1989) reported that after a T2 lesion the middle leg frequently showed unusually long swing movements and “often stepped onto the tibia or femur of the front leg” (p. 116), an observation that we cannot confirm for unrestrained walking stick insects. This difference may have to do with the pattern of more distributed and overall stronger changes in spatial coordination after T2 lesion as reported here. For example, the strong, opposite effects on the working ranges of the ipsilateral front and middle legs (our Figure 7) would have greatly reduced the likelihood of an overstepping middle leg. This is in line with the fact that increased overstepping in Dean’s animals was accompanied by much less divergence of the front and middle leg working ranges.

Moreover, we found strong anterior shifts of the entire working range of the contralateral middle leg (Figure 7D) and an increased step length in both the contralateral middle and hind legs (Table 1). Both of these effects occurred on the intact body side, where Dean (1989) reported effects with opposite shift directions for the contralateral middle leg AEP and PEP, as well as the hind leg AEP (rearward shifts for Dean, his Table 4; forward shifts for us, our Table 1). Assuming that the single major difference between Dean’s and our experimental design concerned the load distribution and load transfer among legs, we propose that the load distribution experienced by the animal substantially affects adjustments in spatial inter-leg coordination. To some extent, this also concerned the temporal coordination, as Dean (1989) did not report a significant change in temporal coordination of the legs on the intact body side. In contrast, we did find significant adjustment in the coupling of the contralateral hind and middle legs (L2 in L3, Figure 9).

Dean (1989) himself noted quite different effects of a T3 lesion in supported and free walking stick insects. His qualitative observations on free walking animals were that “the most obvious effect […] was an apparent weakness in the rear leg, an inability to make a strong swing movement. […] The ipsilateral rear leg spent much of its time extended near its posterior extreme position (PEP) where it dragged along the surface. This leg contributed support to the animal […]. Only when it was unloaded by the other legs could it occasionally make a short swing” (Dean, 1989, p. 107). He described a different behaviour for tethered walking (with reduced load), when the ipsilateral hind leg stepped more regularly.

Our own observations on free walking insects confirm that, after a T3 lesion, the ipsilateral hind leg moved only little. Overall, the difference between Dean’s study and ours appeared less pronounced for the T3 lesion than for the T2 lesion. Much like Dean (1989), we found that the ipsilateral hind leg does not make normal swing movements, although protraction/retraction of the hind leg femur oscillated rhythmically (Figure 3). Nevertheless, these rhythmic movements concern the femur and do not necessarily imply that the hind leg conducted a genuine, active swing movement with each femoral protraction. In principle, part of this movement could be passive, as induced by a lateral pull by the contralateral legs. As the difference between active and passive movement is impossible to tell from our top view videos (see Supplementary Videos), further studies would have to record protractor activity and track the movement of the hind leg tarsus.

Our findings on temporal coordination after T3 lesion largely corroborate Dean’s findings, in that the coordination of the ipsilateral hind and middle legs was hampered, with greatly increased dispersion, albeit little or no effect on mean phase (R2 in R3; Figure 10). Effects of T3 lesion on spatial coordination look fairly similar in both studies (compare Dean’s Table 2 with our Table 2), except for two differences: First we report a lot more effects than Dean (1989); second, Dean reported a slight rearward shift of the contralateral hind leg AEP (intact body side), whereas we found a substantial forward shift, i.e., in the opposite direction, with an associated strong increase of the step length. Thus, as for the T2 lesion effects discussed above, we find that spatial adjustments on the contralateral (intact) side differ between tethered and unrestrained walking.

### Load Transfer in Temporal and Spatial Coordination

Perhaps the most important difference between the results on tethered walking stick insects (Dean, 1989) and ours concerns the somewhat dysfunctional swing movements and the increased frequency of overstepping that were found in tethered but not in unrestrained walking. What is more, our Figures 2, 3 demonstrate clearly that the overall walking behaviour remained functional after connective lesion, as animals were still capable of walking along the same paths and without significant reduction of forward velocity. In fact, the significant increase in sideward velocity after connective lesion (Figure 4) implies that the difference in net translational velocity, i.e., the resultant of forward and sideward translation, would be even smaller than the difference in forward translation alone. Increased sideward translation indicates that connective lesions induced an asymmetry of the lateral forces exerted by the feet during stance. We suggest that this asymmetry is reflected by significant increase of step length on the intact (left) body side, which we found in the two rear legs after T2 lesion (Figure 6A, L2 and L3) and in all three legs after T3 lesion (Figure 6B, L1–L3). On the lesioned (right) body side, step length increased only in the leg immediately anterior to the lesion (R1 in Figure 6A and R2 in Figure 6B), whereas the leg posterior to the lesion took much shorter steps, and the remaining third leg did not change step length at all. The fact that step duration, i.e., cycle period (Figure 5), did not mirror these changes in step length (see Tables 1, 2) suggests that movement velocities must have differed strongly among legs, potentially on a stride-to stride basis. Given the strong variance of step duration (see 95% CI’s and per-animal means in Figure 5), we propose that legs locally adjusted movement velocity to maintain the much more consistent changes in step length and the associated touch-down (Figures 7A, 8A) and lift-off positions (Figures 7B, 8B). This is consistent with the observation that temporal coordination became highly variable for the leg pair immediately adjacent to the lesion (Figure 9: R1 in R2; Figure 10; R2 in R3) while changing only little or not at all in almost all other leg pairs (except contralateral coupling R2 in L2 and ipsilateral coupling of intact L2 in L2 after T2 lesion, Figure 9). Thus, we conclude that animals compensated for hampered inter-leg coordination in a single leg pair by concerted action of all legs, leading to substantial adjustment of spatial coordination with comparatively little change in temporal coordination.

Concerning the contribution of load transfer among legs to temporal coordination according to Cruse’s coordination rule 2, Dallmann et al. (2017) provided strong evidence for two important aspects of mechanical inter-leg coupling: First, local unloading of a middle leg may be related to a single, most likely cause, that is the touch-down of the posterior hind leg (i.e., the sender leg); second, local unloading precedes the switch of depressor to levator activity, i.e., the transition from stance to swing. Assuming that this evidence would hold for the experimental situation of the present study, we expected that normal ipsilateral coordination should have persisted even after connective lesion. This was clearly not the case. Nevertheless, while the fact that both lesions resulted in highly variable phase relationships clearly points at the role of neural information transfer through the ipsilateral connective, the small (Figure 9) or even non-significant (Figure 10) change in mean phase lag among animals indicates the persistence of some weak coordinating effect. Whether or not this weak effect could be driven by ipsilateral load transfer or rather by an influence coming from the intact contralateral leg cannot be decided based on our results. As yet, the results of Dallmann et al. (2017) indicate that mechanical load transfer among two legs may only be effective if the distance between feet is small. Accordingly, our finding of increased distance between the sender leg AEP and receiver leg PEP (Figures 7C,D, 8C,D) adjacent to the lesion should have reduced efficacy of mechanical load transfer and weakened its potential effect on inter-leg coordination.

Mechanical load transfer alone cannot maintain inter-leg coordination after connective lesion in cockroaches either. “In a male *Blatta* with the right pro-mesothoracic commissure cut, the legs of the uninjured side showed a perfect rhythm L3, L2, L1 and this was true of the right side to some extent, but sometimes *R1* fell out of the rhythm” (Hughes, 1957, S. 323). Similarly, Greene and Spirito (1979) found in that slow walking, tethered but load-bearing *Periplaneta americana*, connective lesions caused strong immediate effects on ipsi- and contralateral phase differences in the leg pair posterior to the lesion. Similar to our own results, they found that the main effect concerned the precision of coordination (i.e., strongly increased variance), whereas the mean phase changed relatively little. Moreover, the remaining leg pairs maintained rigid coordination, but with slightly altered mean phase. Quite fitting to our own study, the authors concluded that “it should be stressed at this point that these co-ordination measures are not independent; a change in the relationship between any one pair of legs will necessarily be accompanied by changes in other pairs. Thus, the entire system must be considered as an entity” (Greene und Spirito 1979, S. 251). Overall, due to the extensive adjustments of all legs to a local defect in neuronal information transfer, it would be far-fetched to stress the significance of a single local mechanism of inter-leg coordination. Our results show that stick insects adjust to connective lesion quite differently if they experience a naturalistic load distribution. However, since altered load distribution during walking on inclines causes relatively weak effects on footfall patterns of the legs or body posture, but rather strong effects on muscle activity (Dallmann et al., 2019) future studies may need to relate load-induced changes in distributed muscle activity to ensuing kinematic changes in conjunction with local lesions.

As the experimental situations of Dean’s study (1989) and ours mainly differed in load distribution among legs, we conclude that these differences must be related to load. Future modelling studies using suitable dynamic simulation environments (e.g., see Schilling and Cruse, 2020), biomimetic robots with distributed load sensing (e.g., see Dürr et al., 2019) or conceptual robot models with load-dependent step-cycle generation (e.g., Owaki et al., 2013) could test the main prediction of our study: A change in load distribution (e.g., tethered vs. free walking) can account for compensatory spatial coordination after disruption of information exchange between neighbouring legs, so as to maintain the walking speed before the disruption. A corollary of this prediction is that such spatial compensatory actions occur at the cost of increased step-by-step variation of temporal coordination among legs with disrupted information exchange. Furthermore, we expect to see significant spatial adjustment on the contralateral (intact) side of the disrupted information exchange.

## Data Availability Statement

The raw data supporting the conclusions of this article will be made available by the authors, without undue reservation.

## Author Contributions

MN, MJ, and VD designed research and edited the manuscript. VD established the setup and analysis software, and advised MN and MJ. MN and MJ performed the experiments, analysed the data, prepared all the figures, and drafted the manuscript. VD acquired the funding. All authors contributed to the article and approved the submitted version.

## Conflict of Interest

The authors declare that the research was conducted in the absence of any commercial or financial relationships that could be construed as a potential conflict of interest.
