# Supplementary material for: Effect of Thoracic Connective Lesion on Inter-Leg Coordination in Freely Walking Stick Insects
Source: Front Bioeng Biotechnol. 2021 Apr 20;9:628998. doi: 10.3389/fbioe.2021.628998 (PMC8093632; doi:10.3389/fbioe.2021.628998)
Supplement: Supplementary file 1 [file Data_Sheet_1.PDF]

Supplementary Material

Of the paper entitled "***Effect of thoracic connective lesion on inter-leg coordination in freely walking stick insects***", by Miriam Niemeier, Manon Jeschke, and Volker Dürr (2021).

Contents

Table S1: Trial and step numbers per animal and per cohort 1
Figure S2: Example trial for T2-Lesion (Animal09_170929_02) 3
Figure S3: Example trial for T3-Lesion (Animal12_171002_07) 4
Supplementary methods information and explanation of example files 5

Table S1: Trial and step numbers per animal and per cohort.

Thorax segment specifies the site of the operation (T2: Mesothorax, T3: Metathorax)

Animal	Thorax segment	Treatment	Number of trials	Number of steps
04	T2	Lesion	15	2288
01	T2	Lesion	15	1499
09	T2	Lesion	19	2713
06	T2	Lesion	14	2200
08	T2	Lesion	12	1721
**Total, T2 Lesion**			**75**	**10421**
05	T2	Sham	20	1858
03	T2	Sham	20	1524
07	T2	Sham	14	1252
10	T2	Sham	11	1155
02	T2	Sham	4	442

**Total, T2 Sham**			**69**	**6231**
20	T3	Lesion	20	2487
11	T3	Lesion	14	884
12	T3	Lesion	16	1447
16	T3	Lesion	13	1177
18	T3	Lesion	11	1320
**Total, T3 Lesion**			**74**	**7315**
19	T3	Sham	20	2726
21	T3	Sham	19	2954
14	T3	Sham	18	1837
13	T3	Sham	17	1901
17	T3	Sham	15	1658
**Total, T3 Sham**			**89**	**11076**

Figure S2: Example trial for T2-Lesion (Animal09_170929_02)

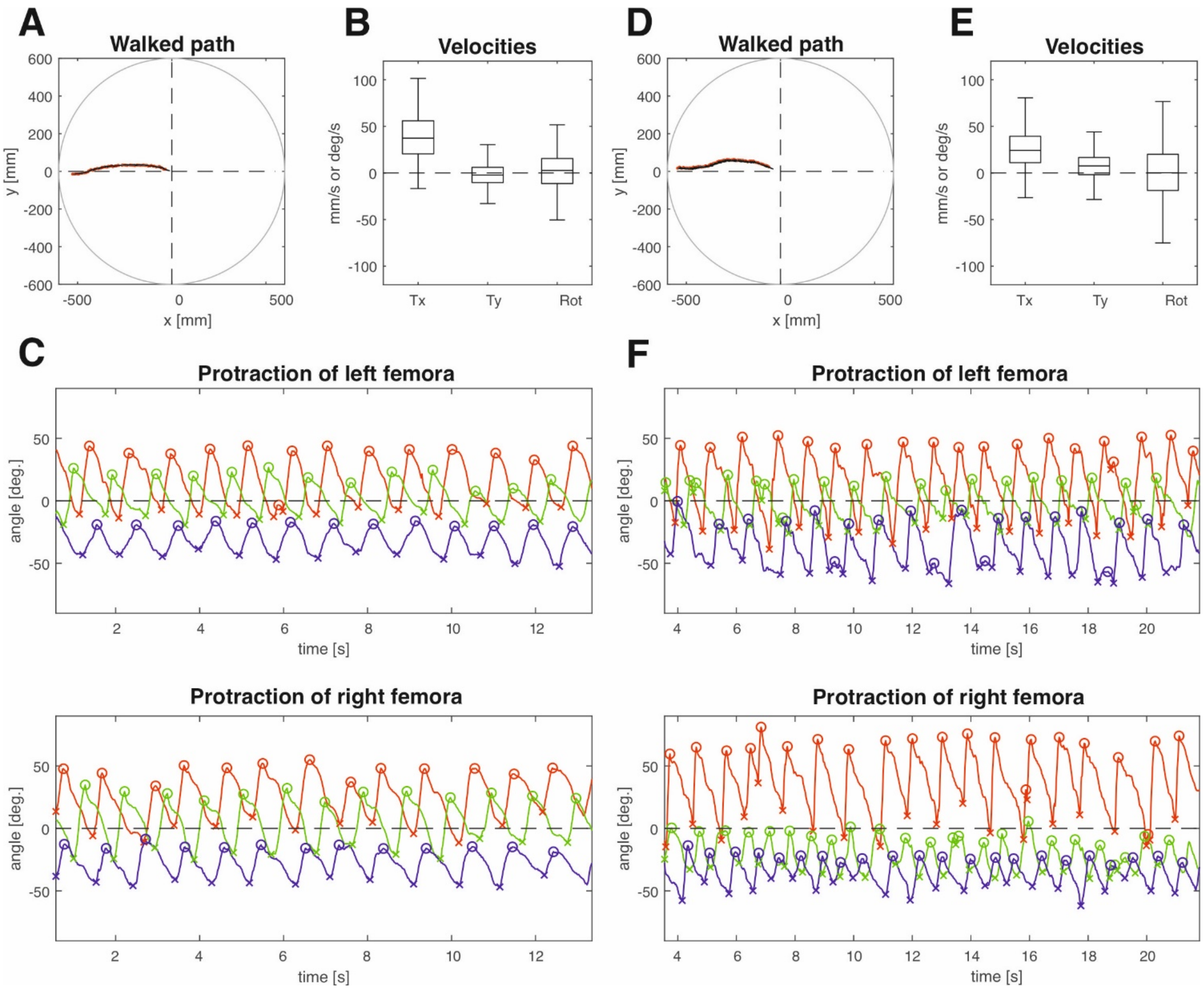

Same figure details as in manuscript Fig. 2, but with entire trial being shown in panel F (which had been truncated in Fig. 2 to achieve same axis scale with panel C).

Figure S3: Example trial for T3-Lesion (Animal12_171002_07)

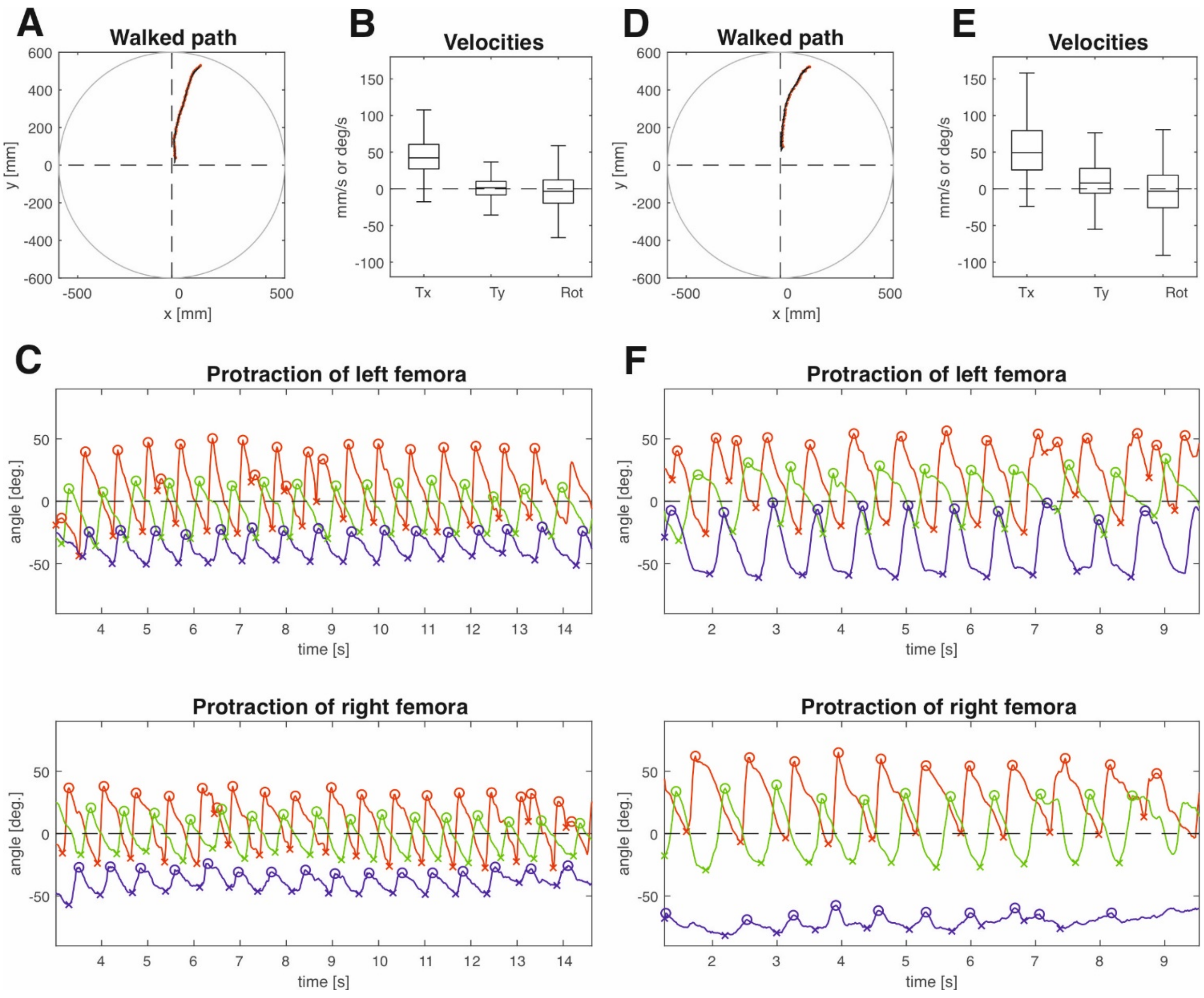

Same figure details as in manuscript Fig. 3, but with entire trial being shown in panel C (which had been truncated in Fig. 3 to achieve same axis scale with panel F).

Supplementary methods information and explanation of example files

As an extension to the method section "Data analysis" of the main manuscript, the Supplementary Material contains video files and processed data files for the four trials shown in Figs 2 and S2 (T2-Lesion: Animal09_170929_02*.*; T2-Sham: Animal07_170928_04*.*), as well as for Figs 3 and S3 (T3-Lesion: Animal12_171002_07*.*; T3-Sham: Animal13_171002_13*.*). Since the original video files had been recorded with infrared flashlight illumination only, they were perfectly suited for semi-automatic marker detection, albeit being dark and contrast-poor for the human eye. Accordingly, brightness and contrast of the video files provided have been adjusted.

The corresponding *Matlab* files contain all original data (except the video), the calibration data, and the processed secondary data of a single trial. These files were generated in three steps: In a first step, the recorded videos were processed (Fig. S4, S5), yielding image positions of the nine markers (Fig. S4) for each video frame. In a second step, the time series of the camera position records from the gantry system (variable *tc* in Fig. S6) and marker trajectories of the video analysis (variable *data* in Fig. S6) were imported, along with calibration files for the camera (variable *calib* in Fig. S6; from Matlab camera calibration toolbox) and the gantry and the arena beneath it (variable *setup* in Fig. S6; from custom-written scripts) containing axis orientation, spatial resolution etc. The third step involved the calculation of all secondary variables shown in Figs S6 and S7.

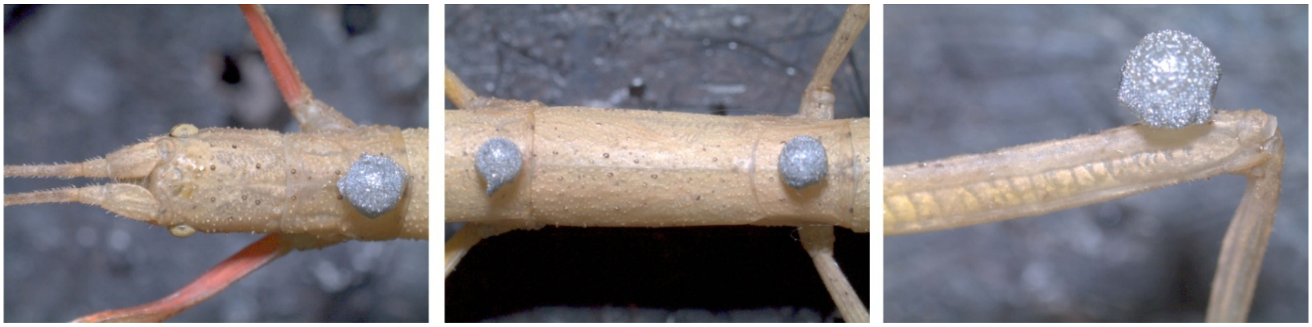

Figure S4: Marker placement on the stick insect. For the marker-based tracking algorithm to work, retro-reflective markers were placed on each one of the three thorax segments, labelling the point between the leg bases (*Left:* T1 marker on prothorax; *Middle*: T2 and T3 markers on mesothorax and metathorax, respectively; note that the metathorax in stick insects is fused with the first abdominal segment, such that the T3 marker is actually fixed to the tergite of the 1st abdominal segment). The thorax markers served as reference points for the body-fixed coordinate system, and for the calculation of the protraction/ retraction angles of the femora. Each one of the six legs carried a marker on the dorsal surface of the distal femur (*Right*). Protraction/retraction angles of the legs were inferred from lines connecting a femur marker to its corresponding thorax marker.

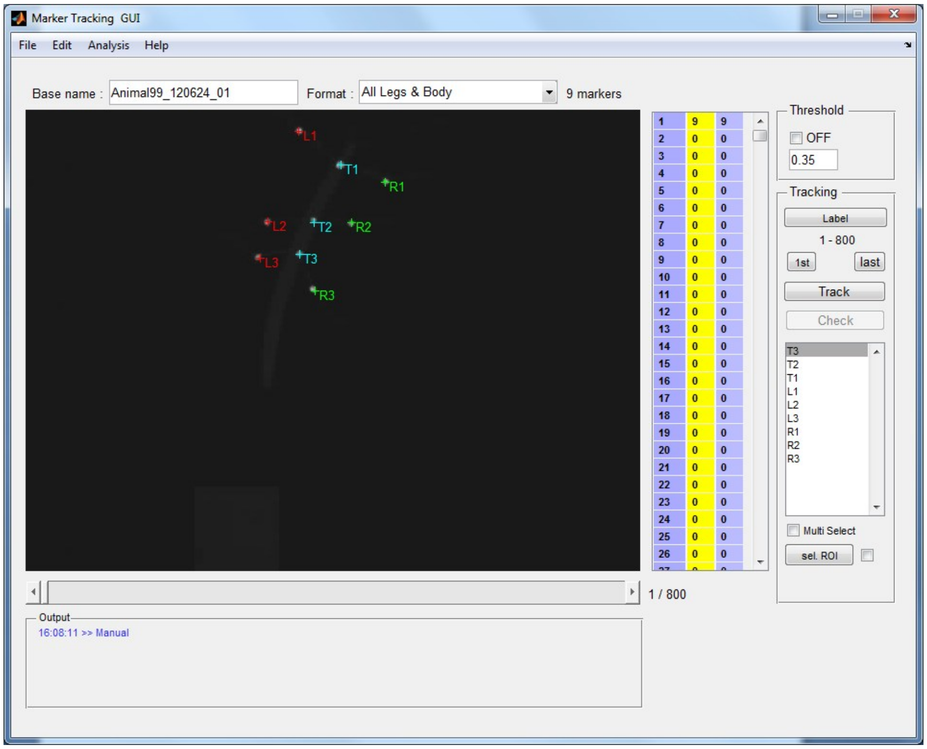

Figure S5: Marker tracking. Videos were loaded into a custom-written graphical user interface (GUI) in Matlab to conduct semi-automatic marker tracking. A threshold level (top right; here 0.35) set the relative brightness threshold Θ that was used to binarize the image into white (if brighter than Θ) and black pixels (if darker than Θ). By use of standard clustering routines of *Matlab*, 2D image positions were estimated as the centre of gravity of each white pixel cluster (potentially, yielding more clusters than markers). Once the user had manually labelled all nine marker positions within the initial frame i_0 of a video episode, a simple nearest-neighbour algorithm was applied to determine which white pixel cluster in the subsequent frame corresponded to the labelled cluster of the previous frame. This automatic assignment could be inspected and manually corrected, if necessary. The right panel of Fig. S7 gives an overview of the data structure generated by the marker tracking GUI.

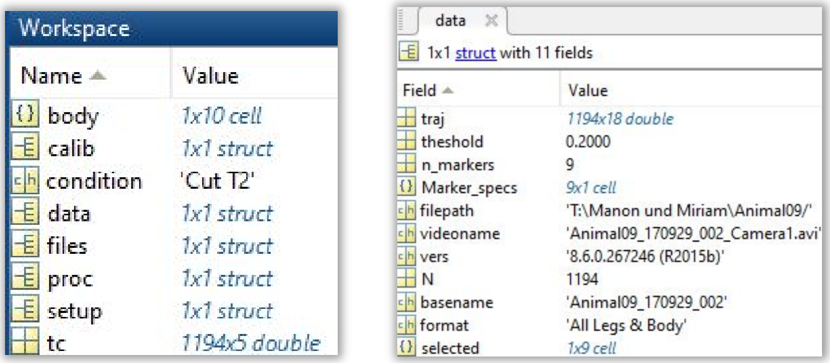

Fig. S6: Workspace (left) and content of marker trajectory variable *data* (right). The main variables of the processed data files were those containing marker position data (*data*), camera position data (*tc*), camera calibration (*calib*), gantry calibration (*setup*) and a large data structure containing all secondary processed data (*proc*). The N×M matrix *data.traj* (top of right panel) contains the xy-pixel coordinates of M/2 markers from N frames. From file: *T2_Lesion_Animal09_170929_002_combined.mat*.

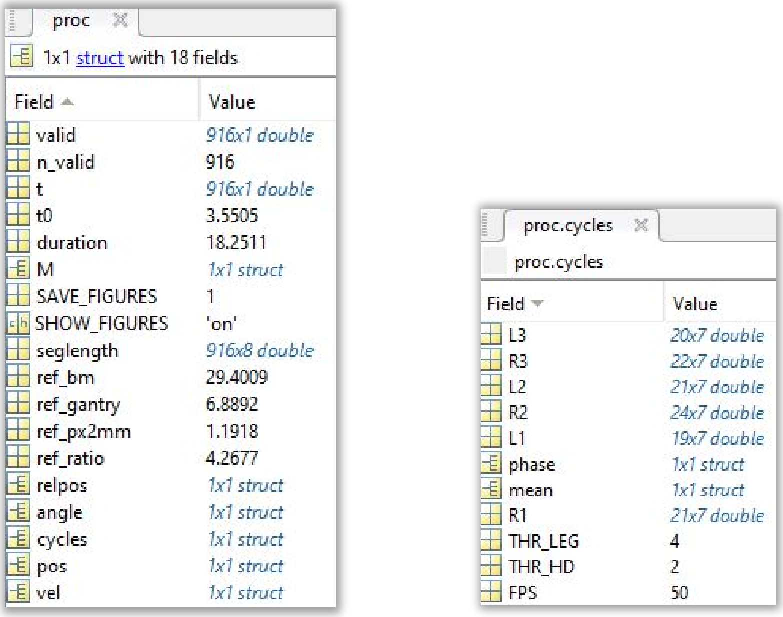

Fig. S7 Content of secondary analysis variable *proc* (left) and the corresponding step cycle variable *proc.cycles* (right). As a first step of the secondary data analysis, marker coordinates were converted into a coordinate system aligned with the gantry coordinate system, and centred on the camera image (*proc.M*). Moreover, the time stamp of each frame was converted to seconds (*proc.t*:), with *proc.t0* indicating the start time of the tracked video sequence (which varied from trial to trial). Then, the camera and marker position coordinates were combined to provide the absolute position of the animal in the arena (in mm), and the three velocity components of the moving animal were calculated for subsequent pairs of frames (*proc.vel*, containing two translational velocities in [mm/s] and yaw rotation in [deg/s]). From file: *T2_Lesion_Animal09_170929_002_combined.mat*.

Next, the body axis orientation within the arena was used to counter-rotate all marker positions so as to indicate the relative positions of each markers in body-centred, metric coordinates (*proc.relpos* in mm). From these, the protraction/retraction angles of the legs were calculated and stored as time courses (*proc.angle* in degrees; zero being perpendicular to the body axis; positive values for anterior positions). Using the function *peakfinder.m* (Version 2009 by Nathanael Yoder; available from the *Matlab Central* server), we then extracted times and magnitudes of the peaks/troughs of the protraction/retraction angle time courses (*proc.cycle*s, Fig. S7, right) and stored them step-by-step for each one of the six legs (R1 to R3 and L1 to L3 as fields of variable *proc.cycle*s). For example *proc.cycles.R3'* is a 3×7 matrix with seven key parameters for three complete step cycles of the right hind leg. The columns of this matrix contained the indices of start (initial lift-off at PEP), mid (touch-down at AEP) and stop (terminal lift-off at next PEP) points of each protraction-retraction cycle, the step period and amplitude in [s] and [deg.], respectively, followed by the median translational and rotational velocities during this step cycle in [mm/s] and [deg./s], respectively. Figures S2 C, F and S3 C, F show the extreme positions as detected by *peakfinder*. For example, the blue circles corresponding to the AEP of the right hind leg (R3) in Fig. S2 F were plotted by use of the variables *proc.t* and *proc.angle.R3*, and the indices stored in *proc.cycles.R3(:,2)*.
